# Dissecting stress with transcriptomics

**DOI:** 10.18632/oncotarget.14693

**Published:** 2017-01-17

**Authors:** Mattia Privitera, Amalia Floriou-Servou, Johannes Bohacek

**Affiliations:** ETH Zurich, Department of Health Sciences and Technology, University of Zurich, Brain Research Institute, Neuroscience Center Zurich, Zurich, Switzerland

**Keywords:** stress, transcriptome, norepinephrine, gene expression, stressome

In our ever-changing environment, we are frequently exposed to uncontrollable, threatening social or physical stimuli that are perceived as stress. The brain rapidly mounts a stress response, by releasing neurotransmitters, hormones and peptides - stress mediators - that orchestrate organism-wide changes in an attempt to meet the challenges of the stressful situation. Despite the adaptive value of this evolutionary conserved response, severe or prolonged stressors are also linked to many psychiatric diseases, most prominently anxiety and depression. Unraveling the molecular mechanisms underlying the stress response thus promises the discovery of new therapeutic targets to prevent or treat stress-related disorders.

Many classic stress mediators have been intensively studied for decades, most prominently corticosterone (CORT), corticotropin-releasing hormone (CRH) and norepinephrine (NE). However, understanding the complexity of the stress response, which involves multiple parallel and converging molecular signaling pathways, still presents a major research challenge. The recent revolution in transcriptomic analyses now allows the generation of detailed, reproducible, genome-wide molecular readouts of stress-induced changes across various tissues [1]. This enables thorough and innovative approaches, which have started to reveal surprising answers to old questions. For example, CORT was long thought to be responsible for most gene expression changes after stress, mainly because the glucocorticoid receptor (GR) acts as a transcription factor. However, when recent studies showed that rapid stress-induced gene expression changes occur in the brain independently of CORT [2-4], it became apparent how little is known about what causes these immediate molecular changes. Our latest research has thus focused on dissecting the early stress response using pharmacologic and transcriptomic tools. We first characterized the transcriptomic changes shortly (45 minutes) after exposure to an acute swim stress challenge. We observed profound transcriptome-wide changes in the hippocampus, a stress-sensitive brain region that expresses high levels of GR and CRH receptors. Pharmacologically blocking either of these receptors hardly had any effect on the observed gene expression changes. However, blocking *β*-adrenergic receptors strongly prevented the stress-induced increase of many genes [4]. Although the role of NE as an important mediator of the stress response has been well known, its strong impact on gene expression changes was novel. In subsequent studies we were able to demonstrate the specific contribution of *β*2-adrenergic receptors to the regulation of individual stress-responsive genes. This demonstrates the versatility of transcriptomic tools to generate fundamental insights into basic biological processes. Together with NE, glutamatergic signaling likely plays a key role in mediating immediate stress-induced transcriptomic changes [5], while CORT is expected to contribute to subsequent waves of gene expression. Therefore, systematically analyzing the transcriptomic impact of several stress mediators across various time points will be necessary for generating a complete molecular picture of the immediate stress response.

Beyond dissecting the rapid molecular changes after stress, transcriptomics can address other key questions in the field of stress research. For example, why are some individuals susceptible to developing neuropsychiatric disorders after traumatic stress exposure, while others are resilient to the same stressor? A transcriptomic approach has recently led to the discovery of a novel set of hippocampal “hub genes”, genes whose experimental overexpression increases stress susceptibility, while their suppression promotes resilience [5]. To fully understand stress vulnerability, it will also be important to apply transcriptomics to studying sex differences, because stress-induced disorders in the clinic show strong sex bias. Accordingly, the transcriptional response to stress is dramatically different between females and males in the hippocampus [3] and the nucleus accumbens, likely due to epigenetic factors [7]. Of course, hippocampus and nucleus accumbens are only part of a larger, complex stress circuitry in the brain, and it will be necessary to compare the transcriptomic response after stress exposure in multiple brain regions simultaneously. One such network-approach has elegantly revealed important functional coupling of stress-induced transcriptomic changes between some regions, and opposing gene regulation between others [6]. Importantly, individual brain regions are extremely heterogeneous tissues, comprised of many different types of excitatory and inhibitory neurons as well as non-neuronal cells. Different neurons have specialized connections and express specific receptors, hence they differ vastly regarding the type of information they receive, their function within local circuits, and the output they send to local or distant targets. The advent of single-cell transcriptomics now allows identifying cell identity with great precision. This approach holds the potential to reveal critical insights into cell-type specific molecular responses to stress in health and disease.

As transcriptomics becomes more affordable and is complemented by proteomics and metabolomics, we envision the establishment of a molecular roadmap of stress - *the stressome*. This database will catalogue stress-induced molecular changes within individual cell types, local circuits, and brain-wide networks, track these changes across time, delineate their dependence on various stress mediators, and serve as a resource and guide to basic research and drug development.
